# The Incidence of Needlestick and Sharps Injuries Among Healthcare Workers in a Tertiary Care Hospital: A Cross-Sectional Study

**DOI:** 10.7759/cureus.38097

**Published:** 2023-04-25

**Authors:** Sarah Alshehri, Malik Kayal, Hawazen Alahmad Almshhad, Qais Dirar, Wael AlKattan, Atef Shibl, Abderrahman Ouban

**Affiliations:** 1 Department of Immunology and Microbiology, Alfaisal University College of Medicine, Riyadh, SAU; 2 Department of Anatomical Sciences, Alfaisal University College of Medicine, Riyadh, SAU; 3 Department of Pathology, Alfaisal University College of Medicine, Riyadh, SAU; 4 Department of Surgery, Alfaisal University College of Medicine, Riyadh, SAU

**Keywords:** needlestick, healthcare worker, precautionary measures, hiv infection, viral hepatitis b and c

## Abstract

Background

Needlestick injuries (NSIs) and sharps injuries (SIs) remain significant hazards in most healthcare facilities that expose healthcare workers (HCWs) to blood-borne pathogens (e.g., HIV, hepatitis B, and hepatitis C). This study aims to review the incidence of NSIs and SIs in King Fahad Medical City (KFMC) and correlate this incidence with several parameters related to the event, including age, sex, length of work experience, type of injury, type of instrument causing the injury, type of activity during which the injury happened, nature of the job of the HCWs, and location within the hospital where the injury happened.

Methodology

This cross-sectional study involves all self-reported documents related to needlestick and sharp injuries among HCWs at King Fahad Medical City (KFMC) in Riyadh, Kingdom of Saudi Arabia, from January 2017 to December 2020. The data of 389 reports of needlestick and sharp injuries detailing incidence and site, shift, type, and instrument related to the incidents were reported to the infection control department for coding and analysis using the Statistical Package for the Social Sciences (SPSS) version 22 (IBM SPSS Statistics, Armonk, NY, USA).

Results

Our data showed that NSIs/SIs could be caused by a wide range of objects used by healthcare workers, including needles, suture needles, scalpels, and sharp devices. Remarkably, the most common cause of NSIs was handling the sharp object (38.8%), followed by disposing of the sharp object (19.3%). Furthermore, nurses were found to be the highest at-risk category of HCWs experiencing NSIs (49.9%), while medical waste handlers (1.5%) and dentists (1.3%) were least likely to incur injuries.

Conclusion

This study sheds some light on the incidence rates of NCIs and SIs at KFMC and correlates these rates with several demographical, occupational, and experiential parameters related to these events.

## Introduction

A needlestick injury (NSI) is defined as an accidental skin-penetrating stab wound from a hollow-bore needle (or any sharp) containing another person's blood or body fluid. Sharps injury (SI) is defined as a skin-penetrating stab wound caused by sharp instruments and accidents in a medical setting [1]. NSIs are more predominant among healthcare workers (HCWs) as they are at a higher risk of exposure to needles due to their clinical practice [2,3]. It is estimated globally that up to 44.5% of healthcare workers report at least one event of accidental NSIs or SIs annually [2,3]. The annual incidence of NSIs among healthcare workers in Saudi Arabia was estimated to be around 22.2% between October and November 2021 and was highest among physicians, followed by nurses [4]. However, it is also estimated that more than half of the injury events were not reported in Saudi Arabia [5]. Also, NSIs risk exposure to more than 20 blood-borne diseases, including HIV, hepatitis B, and hepatitis C [5]. In South Korea, 60 hospitals participated in a study, and it was revealed that due to the absence of safety training programs for HCWs, HCWs are highly likely to incur NSIs/SIs [4].

According to the literature, most NSI/SI incidents occurred during the first year of training (57.57%), which is believed to be due to a lack of training and awareness [6]. The most common place for NSIs was the patient room, followed by the emergency room [7]. Therefore, protection measures should be applied to decrease NSIs/SIs and protect HCWs from unwanted circumstances. Double-gloving has effectively helped in preventing NSIs/SIs. However, it causes discomfort and impaired sensation for HCWs [8], for which blunt needles were developed that require much force to perforate the gloves and cause injury to HCWs preventing NSI occurrence [9]. Furthermore, training HCWs regarding NSIs/SIs is the most effective way of preventing NSIs [10]. In addition, removing any hazard within the practice environment that may influence the risks of incurring NSIs/SIs is another effective way to prevent NSIs/SIs [11].

This study will discuss the incidence of NSIs/SIs among HCWs at a single center, King Fahad Medical City (KFMC), in Riyadh, Saudi Arabia, and will explore the potential risk factors for incurring NSIs and SIs across the different departments and specialties.

## Materials and methods

After obtaining Institutional Review Board (IRB) approval from the institutional ethics committee of King Fahad Medical City, the study was conducted involving all HCWs, who reported NSIs or SIs between 2017 and 2020, targeting areas that intensively use sharp objects, including the operating rooms, emergency rooms, ICUs, surgical departments, dentistry, and laboratory.

An assessment of documents related to NSIs among HCWs at King Fahad Medical City (KFMC) was collated using a cross-sectional study design. The incidence rate of the variable epidemiological outcome (i.e., NSIs and SIs) and the exposure characteristics were measured in all subjects to determine the incidence. Data on NSI incidents at KFMC was gathered by reviewing notification forms completed by HCWs containing types of injuries, causes, and whereabouts in the hospital to be reported to the infection control department of the hospital. All HCWs who experienced NSIs/SIs with complete data between 2017 and 2020 were included in the final sample size, with the exclusion of participants with incomplete data.

Statistical analyses

These included frequency distribution, chi-squared tests, and calculations of NSI/SI rates. A p-value of <0.05 was considered statistically significant. The data of 389 NSI/SI incidents reported to the infection control department at KFMC were coded and entered into a spreadsheet program, before being analyzed using the Statistical Package for the Social Sciences (SPSS) version 22 (IBM SPSS Statistics, Armonk, NY, USA). The prevalence of the incidence of NSI/SI concerning personal and work characteristics and related NSI/SI factors was calculated. The chi-squared test was used to study the association between NSIs/SIs with infected instruments and the different characteristics of NSIs/SIs among HCWs to identify the corresponding risk factors. Finally, logistic regression analysis was used to estimate the odds ratio (OR) and 95% confidence interval (95% CI) for NSIs/SIs with infected instruments related to different factors.

## Results

The specific objectives were to calculate the incidence of NSIs/SIs among HCWs and identify its risk factors at KFMC from 2017 to 2020.

The study findings revealed that 6.9% of HCWs reported that it was not their first NSI, while most NSI incidents (93.1%) occurred with HCWs for the first time. In addition, most NSI incidents occurred with HCWs immunized against hepatitis B, while only 17.5% occurred with non-immunized workers. Furthermore, the majority of injured HCWs (92.3%) reported that they took sufficient precautions to avoid NSIs, while only 7.7% of those injuries were correlated with HCWs with no sufficient precautions.

Table 1 highlights the incidence of NSIs with infected needles and sharps by personal, work, and NSI characteristics. The chi-squared test results were used to examine the association between NSIs with contaminated instruments and personal, work, and NSI characteristics.

**Table 1:** Comparison of NSIs with infected instruments by personal, work, and NSI characteristics *Significant association at 0.1 level **Significant association at 0.05 level NSI: needlestick injury, ICU: intensive care unit

Characteristics		Frequency	%	Infected	p-value
Gender	Female	264	67.9	13 (4.9%)	0.006**
	Male	125	32.1	16 (12.8%)	
Age	21-30 years	221	56.8	12 (5.4%)	0.08*
	>30 years	168	43.2	17 (10.1%)	
Experience	Up to 10 years	353	90.7	23 (6.5%)	0.03**
	More than 10 years	36	9.3	6 (16.7%)	
Instrument type	Needle	336	86.4	20 (6%)	0.004**
	Scalpel	14	3.6		
	Instrument	8	2.1		
	Suturing needle	4	1		
	Other	27	6.9		
Place of injury	Patient's room	143	36.8	7 (4.9%)	0.279
	Operating room	64	16.5	7 (10.9%)	
	ICU	74	19	8 (10.8%)	
	Other	108	27.7	7 (6.5%)	
Job	Physician	68	17.5	18 (26.5%)	<0.001*
	Nurse	194	49.9		
	Technician	44	11.3		
	Intern	35	9		
	Housekeeping staff	27	6.9%		
	Laboratory staff	10	2.6%		
	Medical waste handler	6	1.5%		
	Dentist	5	1.3%		
Shift of injury	Day shift	273	70.2	21 (7.7%)	0.785
	Night shift	116	29.8	8 (6.9%)	
Activity when injured	Suturing	46	11.8	8 (17.4%)	0.007**
	Handling instruments	151	38.8	13 (8.6%)	
	Collecting waste	35	9		
	During injection	35	9		
	Recapping needles	29	7.5		
	Restless patient	10	2.6		
	Passing sharps	8	2.1		
Nature of injury	Needle puncture	321	82.5	20 (6.2%)	0.046**
	Other	68	17.5	9 (13.2%)	
Year	2017			10 (8.5%)	0.632
	2018			10 (9.3%)	
	2019			5 (6%)	
	2020			4 (4.9%)	

Table 2 reports the odds ratio with a 95% confidence interval calculated using logistic regression to study the strength of the association between NSIs with infected instruments and other factors.

**Table 2:** Risk factors of NSIs with infected needles and sharps *Significant association at 0.1 level **Significant association at 0.05 level NSI: needlestick injury, OR: odds ratio, CI: confidence interval

Characteristics		OR	95% CI for OR	p-value
Gender	Female	Reference		
	Male	2.83	1.32-6.09	0.008**
Age	21-30 years	Reference		
	Older than 30 years	1.96	0.91-4.23	0.086*
Experience	Up to 10 years	Reference		
	More than 10 years	2.87	1.08-7.59	0.034**
Job	Physician	10.15	4.53-22.75	<0.001**
	Other	Reference		
Instrument type	Needle	Reference		
	Other	3.23	1.39-7.54	0.007**
Nature of injury	Needle puncture	Reference		
	Other	2.30	1-5.29	0.051*
Activity when injured	Suturing	4.84	1.71-13.7	0.003**
	Handling instruments	2.17	0.87-5.37	0.095*
	Other	Reference		

**Figure 1:**
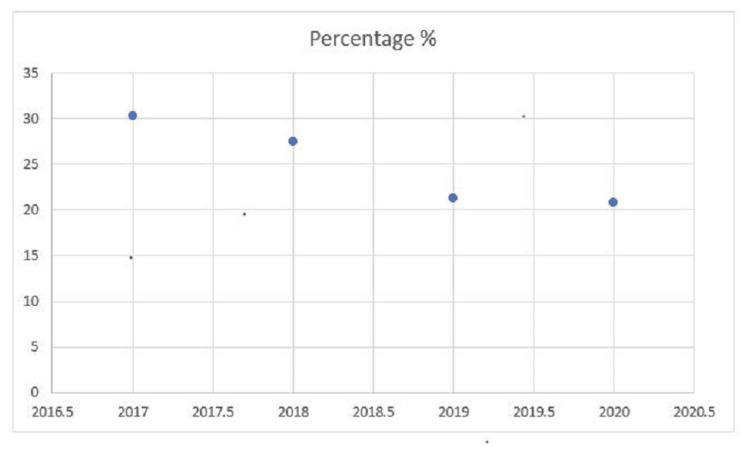
Distribution of NSI incidents NSI: needlestick injury

The distribution of NSI incidents in Figure 1 showed a decline over the study period; the highest rate of injuries occurred in 2017, with 30.3% of the total injuries during the study period, and this percentage gradually decreased to 20.8% by 2020.

## Discussion

This study was conducted at King Fahad Medical City (KFMC) to assess the incidence and other related factors of needlestick injury among healthcare workers from January 2017 to December 2020. Our results indicate that most healthcare workers exposed to NSIs were females, which corroborated recent studies from Saudi Arabia [12,13]. In our study, this finding is most likely related to the fact that most HCWs who reported NSI and SI events were nurses, and the majority of those nurses were females. In addition, the results of our study indicate that the incidence of NSIs was highest among young HCWs (21-30 years), which also corroborates other recent study results [13,14]. However, NSI incidents have decreased in older age groups, which could be related to experience.

In our study, 74% of NSIs have occurred among healthcare workers with less than six years of experience, which correlates with NSI incidents during the first year of training (57.57%) that are believed to be due to a lack of training and awareness [6]. Furthermore, the rate of NSIs in developing countries such as Saudi Arabia is considered high for several reasons, including inadequate training [15]. In our study, the incidence of NSI was highest among nurses compared to physicians; this finding was similar to other studies [7,12,16]. In our institution, this may be explained by the fact that nurses in KFMC outnumber physicians by a ratio of 6:1. It also could be explained by being more exposed to NSIs and SIs, because nurses use and handle the instruments that may cause these types of injuries far more than any other HCW groups. In contrast, one study that evaluated the annual incidence of NSIs among healthcare workers in Saudi Arabia estimated it to be around 22.2% between October and November 2021 and had the highest NSI incidents among physicians compared to nurses [14]. Our study indicated that the NSI percentage had dropped by around 10% from 2017 to 2020. Another significant finding in our study was that more than one-third of NSI has occurred in a patient room and not in an acute setting area, corroborating previous studies [7]. This may be explained by the fact that HCWs in the acute setting are better trained than those in the wards [7]. Finally, our study has revealed a high incidence of participants who took necessary precautions, such as full immunization against hepatitis B, which may indicate rising knowledge of NSIs and SIs in HCWs in Saudi Arabia. This finding was reported previously [12]. On the other hand, other studies reported failure to complete prophylactic vaccines [16,17].

Limitations of the study

One limitation of our study that should be mentioned is the fact that it is a single-center study and, as such, may not be wholly representative of the Kingdom of Saudi Arabia. Future studies should also focus on assessing the education and training programs designed to decrease NSI/SI incidence, as they may provide much-needed help in addressing these injuries [18]. Another important aspect that should be addressed by future studies is the psychological impact of NSIs/SIs sustained by HCWs. Recent studies reported significant effects on mental health in HCWs who sustained NSIs/SIs [19-21].

## Conclusions

Needlestick injuries continue to pose serious health risks to healthcare professionals worldwide. Several reasons for NSI and SI incidence include poor attitudes toward risk prevention and management, inadequate implementation of appropriate precautionary measures, and poor training and knowledge of NSIs/SIs and their preventive measures, among others.

This study has shown that the incidence of NSIs/SIs at KFMC is significantly high, and more precautionary measures ought to be implemented. In addition, supervision of entry-level HCWs is also essential as more experience would be required to avoid NSI further.

Notably, our study reported a wide range of factors that may be linked to the incidence of NSIs among HCWs in the country. These include the number of years of working experience, types of objects used, discipline within the healthcare sector, and location of the work area. Since the risks associated with NSI are dire, including infection, morbidity, and even mortality, proper preventive measures must be put in place at all levels to protect the safety of HCWs while performing their roles in hospitals. These preventive measures should target the critical areas of vulnerability, including the years of working experience, types of objects used, discipline within the healthcare sector, and the area of work.
